# Psychometric assessment and exploratory scale refinement of the Generalized Anxiety Disorder 7-item scale among adolescents and young adults in a Swedish context

**DOI:** 10.1186/s12888-026-08423-0

**Published:** 2026-07-23

**Authors:** Emma Ahlqvist Lindqvist, Magnus Johansson, Emma Hovén, Andreas Frick, Hanna Ljungvall, Pernilla Åsenlöf

**Affiliations:** 1 Department of Women’s and Children’s Health, Physiotherapy and Behavioral Medicine, Uppsala University, Uppsala, Sweden; 2 Centre for Psychiatry Research, Department of Clinical Neuroscience, Karolinska Institutet, Stockholm, Sweden; 3 Centre for Women’s Mental Health, WOMHER - Uppsala University, Uppsala University, Uppsala, Sweden; 4 Department of Medical Sciences, Experimental Cognitive and Affective Neuroscience Lab, Uppsala University, Uppsala, Sweden; 5 Department of Psychology, Uppsala University, Uppsala, Sweden; 6 Centre for Medical Humanities, Department of History of Science and Ideas, Uppsala University, Uppsala, Sweden; 7 Department of Social Work, Uppsala University, Uppsala, Sweden; 8 Geriatrics, Rehabilitation Medicine, and Pain Centre, Uppsala University Hospital, Uppsala, Sweden

**Keywords:** Anxiety, GAD-7, Mental health, Adolescents, Young adults, Psychometrics, Rasch

## Abstract

**Background:**

The Generalized Anxiety Disorder 7-item scale (GAD-7) could provide currently lacking data on anxiety levels among adolescents and young adults in Sweden. However, psychometric assessments of the scale are scarce in this population. Additionally, the structural validity of the scale needs to be examined further, given conflicting findings in other populations. The aim of this study was to psychometrically evaluate the GAD-7 among 15–29-year-olds using Rasch analysis and confirmatory factor analysis in a Swedish, general population sample. In addition, an iterative process explored if revised versions of the scale could be identified that fit the Rasch model in this sample.

**Methods:**

A random, age- and sex-stratified sample of 15–29-year-olds from the Swedish population register was drawn, from which stratified groups were randomly invited to participate. Data (*n* = 590) were collected through a study-specific app. Rasch analyses included investigation of item fit, local independence, principal components analysis of standardized Rasch model residuals, response category functioning, measurement invariance, scale-to-sample targeting, and reliability. In addition, confirmatory factor analyses (CFA) were conducted and interpreted using dynamic, data-adaptive cutoffs. An exploratory, iterative process was undertaken aiming at scale refinement based on results for the GAD-7.

**Results:**

No Rasch analyses provided support for the unidimensionality of the GAD-7, which also displayed issues with disordered response categories and measurement invariance. The CFA found substantial misfit for the one-factor model. The exploratory scale refinement resulted in two abbreviated scales warranting further investigation: Items 1–3 fit the Rasch model as a unidimensional scale and demonstrated acceptable to good reliability. Items 5–7 had good fit after merging the two highest response categories, although demonstrated poor reliability.

**Conclusions:**

No analyses provided support for the unidimensionality of the GAD-7 among 15–29-year-olds in Sweden. While these findings do not support the use of a single sum score, they require confirmation in independent samples. Future research should evaluate whether the abbreviated, candidate scale comprising items 1–3 could be useful to assess anxiety in this population, including examination of criterion validity and the potential benefits of adding a response category to enhance both reliability and scale-to-sample targeting.

**Supplementary Information:**

The online version contains supplementary material available at 10.1186/s12888-026-08423-0.

## Background

The World Health Organization ranks anxiety disorders as the ninth most health-related cause of disability worldwide [1]. Despite this, epidemiological studies investigating anxiety prevalence are rare [2]. In Sweden, available reports point to high levels of self-reported anxiety among adolescents and young adults (AYAs) [3, 4], yet these estimates are largely based on unvalidated, single-item surveys. Studies assessing anxiety in this population using more comprehensive, psychometrically established instruments are lacking [5]. Addressing this gap requires that measurement instruments are validated in the specific sample and context in which they are intended to be used.

The Generalized Anxiety Disorder 7-item scale (GAD-7) [6] is one of the most widely used instruments for assessing anxiety symptoms in both research and clinical practice. Originally developed by Spitzer et al. in the United States in 2006, it was designed primarily as a screener for probable generalized anxiety disorder (GAD), though it has since demonstrated broader utility as a measure of anxiety symptom severity [7]. Its brevity, accessibility, and recommended use by the International Consortium for Health Outcomes Measurement [8] make it a strong candidate for assessing anxiety in Swedish AYA populations. However, psychometric assessments of the GAD-7 are scarce in Swedish samples, and it is not sufficient to assume that validity evidence established in other populations [9] transfers to a new context.

Furthermore, while many studies have supported the proposed one-factor structure of the GAD-7 [9], others have detected problems with the one-factor model fit [10, 11], which raises concerns about the structural validity of the scale. If unidimensionality cannot be confirmed, the use of a single sum score as a proxy for anxiety severity becomes psychometrically questionable. Establishing unidimensionality is therefore a prerequisite for justifying GAD-7 sum score use.

A related methodological concern applies to much of the published psychometric literature on the GAD-7. Rule-of-thumb cutoffs to evaluate model fit indices, such as those proposed by Hu and Bentler [12], have dominated the field despite several published papers documenting that they lack generalizability across different sample sizes, factor loadings, and numbers of indicators [13, 14]. This means that prior psychometric studies may have reached misleading conclusions due to misapplied cutoffs [13]. Recent developments in freely available software now allow analysts to derive sample- and model-specific fit cutoffs in both confirmatory factor analysis [15–17] and Rasch Measurement Theory [18], making it possible to evaluate model fit with greater precision than was previously feasible. Given that published psychometric research on the GAD-7 has largely relied on rule-of-thumb cutoffs, re-evaluation using adaptive, sample-specific cutoffs is warranted.

Within Rasch Measurement Theory (RMT), data are compared to the Rasch measurement model, and misfit indicates that the data may not be suitable for measurement [19]. Unidimensionality, local independence, ordered response categories, and invariance are considered prerequisites for sum scores to adequately represent the intended construct [20, 21]. When data do not fit the Rasch model, it is common practice to undertake an exploratory, iterative process to identify alternative item combinations with better measurement properties. Applying RMT to the GAD-7 in a Swedish AYA sample could help establish to what extent a quantitative conceptualization of anxiety has been operationalized successfully in the scale, and, if necessary, explore refinements that could potentially improve the assessment.

Taken together, the lack of psychometric studies of the GAD-7 in a Swedish context, the uncertain dimensionality of the scale, and the limitations of prior analytic approaches warrant further investigation. The aim of this study was to psychometrically evaluate the GAD-7 among 15–29-year-olds in Sweden using confirmatory factor analysis and Rasch analysis to examine several aspects of dimensionality, response category functioning and measurement invariance, as well as scale-to-sample targeting and reliability where unidimensionality was supported. Where model fit was unsatisfactory, an exploratory iterative process was undertaken to identify improved item solutions.

## Methods

This psychometric study has been approved by the Swedish Ethical Review Authority (Approval number: 2023-05652-02).

### Setting and sample

This study (*n* = 590) is part of a cohort conducted by the interdisciplinary research project UPIC (Young People’s Mental Health in Focus), investigating mental health among 15–29-year-olds in Sweden. A random, age- and sex-stratified selection of 40,000 individuals aged 15 to 29 in 2023 was drawn from the total Swedish population register [22]. From these individuals, two new random, age- and sex-stratified selections of 9000 individuals were drawn in September and November 2024, respectively. These potential participants were invited to join the cohort via postal letter. Postal addresses were obtained from the Swedish State Personal Address Register. Interested persons were encouraged to download a study-specific smartphone app to start the recruitment process, manually or by using the QR code provided in the invitation. Written information about the study and contact information to the research group was available in the invitation as well as in the app. Participants registered for the study via the app using their Swedish Social Security number, followed by verification by BankID or electronic signature (for participants who did not have BankID). All participants had to give written consent electronically before participation. The current study is based on the available baseline data collected in the cohort as of May 2025.

### Procedure and data collection

In the UPIC study app, participants were able to respond to several questionnaires, including the GAD-7. Individual characteristics and sociodemographic data including, for example, age, gender, civil status, country of birth, education, sickness absence and income were obtained through a survey.

#### Generalized Anxiety Disorder 7-item scale (GAD-7)

The current study used the Swedish version of the GAD-7 presented in Table 1. This version is slightly modified from the linguistically validated Swedish GAD-7 provided on the Patient Health Questionnaire Screeners website [23], following comprehension issues regarding item 6 detected in a parallel, qualitative study. Items were presented one at a time in the app. The scale consists of seven items where respondents are asked how often during the last 14 days they have been bothered by seven problems. Response options are “Not at all” (= 0 points), “Several days” (= 1 point), “More than half the days” (= 2 points), and “Nearly every day” (= 3 points), producing ordinal data with a raw sum score ranging from 0 to 21 points. Based on normative data from a general population sample aged 14–92 years (*n* = 5030), it has been recommended that GAD-7 scores ≥ 10 be interpreted as “yellow flags” for the presence of, and need for further evaluation for, any anxiety disorder [24]. Further, 5, 10 and 15 points have been suggested as representing mild, moderate and severe levels of anxiety [6].

**Table 1:** The Generalized Anxiety Disorder 7-item scale: items 1–7

	English original	Swedish version
Introductory text	Over the last 2 weeks, how often have you been bothered by the following problems?	Under de senaste 14 dagarna, hur ofta har du besvärats av följande problem?
Item		
1	Feeling nervous, anxious or on edge	Känt dig nervös, ängslig eller väldigt stressad
2	Not being able to stop or control worrying	Inte kunnat sluta oroa dig eller kontrollera din oro
3	Worrying too much about different things	Oroat dig för mycket för olika saker
4	Trouble relaxing	Haft svårt att slappna av
5	Being so restless that it is hard to sit still	Varit så rastlös att du har haft svårt att sitta still
6	Becoming easily annoyed or irritable	Lätt blivit irriterad eller retlig (Unmodified version: Blivit lätt irriterad eller retlig)
7	Feeling afraid as if something awful might happen	Känt dig rädd för att något hemskt skulle hända
Response options	0 = Not at all 1 = Several days 2 = More than half the days 3 = Nearly every day	0 = Inte alls 1 = Flera dagar 2 = Mer än hälften av dagarna 3 = Nästan varje dag

English original and Swedish version used in this study. Items presented one by one in the study-specific app with the introductory text presented for each item

### Data analyses

The Rasch Partial Credit Model (PCM) for polytomous data with Conditional Maximum Likelihood was applied as the GAD-7 uses multiple ordered response categories [25]. This study aimed to evaluate measurement properties related to dimensionality, response category functioning, measurement invariance, scale-to-sample targeting, and reliability [20, 26]. Unidimensionality, local independence, ordered response categories, and invariance were considered prerequisites for ordinal GAD-7 sum scores to be sufficient for representing a latent measure of anxiety. Reliability and targeting were considered contingent upon these criteria and were therefore only reported for item combinations fulfilling them [20].

#### Dimensionality

Unidimensionality is crucial in Rasch analysis to ensure that the scale adheres to the fundamental assumption of measuring a single underlying construct [20]. Several tests were used to investigate dimensionality. Conditional item infit, i.e. weighted mean squares (MSQ), was investigated to detect possible item misfit to the Rasch model [18, 27], using a simulation-based method to determine cutoff values. Observed item fit statistics were compared to distributions generated from 300 simulated datasets, with each simulation based on the item and person parameters estimated from the empirical data, providing unique infit thresholds for each item. Values above the individual thresholds (i.e. underfitting items) were interpreted as indicative of problems such as multidimensionality, disordered response categories, or poorly formulated items (“noise”). Values below (i.e. overfitting items) were instead interpreted as too predictable responses not contributing enough useful information [20].

Item fit was further investigated by calculating item-restscore associations, i.e. the observed and expected association between an item and a score based on the rest of the items, assessed using Goodman-Kruskal’s gamma. In this case, a lower than expected observed correlation value indicates underfit, whereas a higher than expected observed correlation value indicates overfit. Items were defined as misfitting when the adjusted p-value was ≤ 0.05 [28].

Local independence was assessed by examining residual correlations between item pairs using Yen’s Q^3^ residuals [29] to ensure that items were related to each other only through the latent variable. Local dependence, i.e. items being linked such that responses on one item strongly correlate with responses on another item (strong residual correlations), was interpreted as a risk of inflated reliability estimates, questionable construct validity and bias in sum score estimates [30, 31]. Relative cutoffs for residual correlations to indicate local dependence were set by running simulations with 400 iterations, using the 99th percentile from the simulations [29]. Item pairs with residual correlations exceeding the relative cutoff were interpreted as having local dependence.

A Principal Component Analysis of standardized Rasch model residuals (PCAR) was conducted, applying a parametric bootstrap function with 400 iterations to determine a cutoff for the largest eigenvalue to support unidimensionality. Further, standardized loadings on the first residual contrast factor were examined visually in combination with item locations to see if patterns or deviations were apparent [20].

To further explore the dimensionality and provide comparison results using similar methods to previously published psychometric analyses of the GAD-7, confirmatory factor analyses (CFA) were conducted. The model was estimated using Weighted Least Squares Mean and Variance Adjusted (WLSMV) appropriate for ordinal data [32]. Dynamic fit index cutoffs [33] were produced for the Comparative Fit Index (CFI), the Root Mean Square Error of Approximation (RMSEA) and the Standardized Root Mean Square Residual (SRMR), based on 500 simulations. These were compared to empirical fit indices to judge model fit, aiming for the empirical SRMR and RMSEA to be lower, and the empirical CFI to be higher, than the dynamic cutoffs. If the empirical fit index for SRMR and RMSEA are below the Level-0 cutoffs and CFI is above the Level-0 cutoff, it means the model is within sampling error or fitting perfectly. The cutoffs for Levels 1–3 represent increasingly higher levels of misspecification in the model [33].

#### Response category functioning

Category function assessment aimed for the response categories to be monotonically consistent and logically ordered, relative to the latent variable [34]. Item probability curves were visually inspected to determine whether all response categories had the highest probability of being chosen at some point, indicative of them providing meaningful information. Item threshold locations, i.e., the point on the latent trait continuum where a respondent has an equal probability of choosing either of the two adjacent categories, were examined through item hierarchy plots to see whether the thresholds of an item had ordered locations clearly separated from one another.

#### Measurement invariance/differential item functioning

To assess measurement invariance, potential differential item functioning (DIF) was analysed to evaluate whether the scale performs similarly across sex and age groups. A difference of more than 0.5 logits in average item locations was used as the cutoff for DIF [35]. For age, a model-based recursive partitioning was used, testing different ways to partition the continuous age variable to detect potential DIF [36].

#### Targeting

To evaluate how well the scale was matched to the sample, scale-to-sample targeting was examined through a person-item map. The map was visually inspected, aiming for similar distributions of person and item threshold locations on the logit scale, as well as absence of gaps for threshold locations [37]. The information items can provide about a respondent is contingent upon the number of thresholds close to the respondent’s location. Hence, gaps and uneven distributions were interpreted as poor targeting and reduced reliability for persons located in those areas. Additionally, targeting was inspected by calculating means and standard deviations for both person locations and item threshold locations [20].

#### Reliability

Reliability of person estimates was evaluated using three indices which were considered complementary rather than prioritized hierarchically. The Person Separation Index (PSI) [38] provided a traditional Rasch-based estimate of how well the scale differentiates between respondents with different levels of the latent trait. It is important to note that the PSI excludes participants with minimum or maximum score, which sometimes leads to metrics that differ from other methods. The Expected A Posteriori (EAP) reliability [39, 40] offered a model-based estimate of the precision of person measures, reflecting the proportion of true variance relative to total variance in the posterior distributions (i.e., the range of plausible values for that respondent’s latent score). Finally, a novel Bayesian procedure was used to establish the relative measurement uncertainty (RMU) [41]. This approach draws pairs of samples from each respondent’s posterior distribution and calculates the correlation between the two draws. The mean correlation was estimated using 4,000 plausible values and was presented along with the 95% highest density continuous interval (HDCI). All indices range from zero to one and were interpreted using conventional thresholds of < 0.70 (poor), ≥ 0.70 (acceptable), and > 0.80 (good) reliability [42].

#### Iterative process

As the above described analyses for the GAD-7 indicated severe problems in all areas of validity, an iterative process was undertaken to explore whether alternative item combinations could be identified that fit the Rasch measurement model in the current sample. Focus was on identifying a unidimensional scale guided primarily by conditional item fit and item-restscore, applying the same thresholds as described in the respective analysis sections above. The process started with removing the most underfitting items from the GAD-7 one at a time and rerunning analyses. The deletion of items stopped when no items were misfitting. Local independence, PCAR and DIF were analysed in parallel with conditional item fit and item-restscore for all tested item combinations and all analyses yielded consistent results. Hence, when conditional item fit and restscore indicated unidimensionality for an item combination, the other analyses also showed satisfactory results. These item combinations were then tested for response category functioning, where detected issues prompted further refinement before rerunning analyses. The iterative process thus continued until an item combination was identified which had support for unidimensionality and local independence, was measurement invariant and showed satisfactory response category functioning. At this stage, targeting and reliability were assessed. To investigate whether the underfitting items from the GAD-7 could similarly be reduced to a unidimensional scale, the same process was applied in reverse, systematically removing overfitting items from the GAD-7.

The exploratory nature of the iterative refinement process carries a risk of overfitting, as the abbreviated scales were derived from the same dataset used for the initial evaluation of the GAD-7. To assess the stability of item fit results and the risk of overfitting, a 10-fold cross-validation of conditional item infit was conducted for the two candidate abbreviated scales (items 1–3 and items 5–7) separately. The dataset was randomly split into 10 folds of approximately 59 participants each, with each iteration using 9 folds to estimate item infit and generate simulation-based cutoff values based on 300 simulations. This was repeated across all 10-fold combinations, yielding a distribution of infit MSQ values and adaptive cutoffs for each item, thereby allowing assessment of whether item fit results were consistent across subsamples. In addition, item-restscore analyses were conducted for both abbreviated scales using 300 non-parametric bootstrap iterations (sampling with replacement from the complete response data), to obtain probability estimates of item misfit.

#### Supplementary, descriptive analyses

For descriptive purposes, person fit and ordinal sum scores for the sample were calculated and are presented in Additional File 1. Person fit was examined using the non-parametric U3 statistic generalized to polytomous items [43], flagging respondents who did not fit the Rasch model. Item parameters and a transformation table of ordinal sum scores to corresponding interval scores are available in Additional File 2.

#### Software used

All analyses were conducted using the software R, version 4.5.1 [44]. The package *easyRasch* [45] was used for the Rasch analyses, applying *eRm* [46], *mirt* [47], *psychotree* [36, 48], *iarm* [49] and *catR* [50]. The *lavaan* package [51] was used for the CFA with dynamic cutoffs generated using *dynamic* [52]. Person fit to the Rasch model was assessed with *PerFit* [53].

## Results

### Participants

In May 2025, 591 15–29-year-olds had responded to the GAD-7 in the UPIC cohort baseline data collection. One participant was excluded from analyses due to incomplete responses to the GAD-7. The remaining 590 participants had responded to all GAD-7 items. All ages between 15–29 were represented and the median age was 22 (Q1–Q3: 18–26). Sample characteristics are presented in Table 2.

**Table 2:** Sample characteristics

	Frequency (Percentage)
**Sex** (according to the Swedish population register)	
Female	411 (69.7)
Male	179 (30.3)
**Gender** (self-reported)	
Woman	351 (59.5)
Man	161 (27.3)
Non-binary	11 (1.9)
Other	1 (0.2)
Do not know/Do not want to answer	5 (0.8)
Missing data	61(10.3)
**Country of birth**	
Sweden	484 (82.0)
Other Nordic country	0 (0.0)
Other European country	20 (3.4)
Country outside of Europe	24 (4.1)
Missing data	62 (10.5)
**Highest completed education**	
Have not completed primary school	37 (6.3)
Nine-year compulsory school	142 (24.1)
2–4-year upper secondary school or vocational school	220 (37.3)
University or college	108 (18.3)
Folk high school or equivalent	17 (2.9)
Other	5 (0.8)
Missing data	61 (10.3)
**Occupation** (multiple choice possible)	
Full-time employee	120 (20.3)
Part-time employee	93 (15.8)
Student	309 (52.4)
On parental leave	8 (1.4)
Job seeker	61 (10.3)
On sick leave	23 (3.9)
Missing data	63 (10.7)
**Living with parents**	
Yes	298 (50.5)
No	231 (39.2)
Missing data	61 (10.3)
**Relationship status**	
Live together with a partner	134 (22.7)
Have a partner but do not live together	103 (17.5)
Do not have a partner	282 (47.8)
Do not know/do not want to answer	4 (0.7)
Other	6 (1.0)
Missing data	61 (10.3)
**Ever got a psychiatric diagnosis**	
No	26 (4.4)
Yes	134 (22.7)
Missing data	430 (72.9)

### Assessment of the GAD-7

**Fig. 1:**
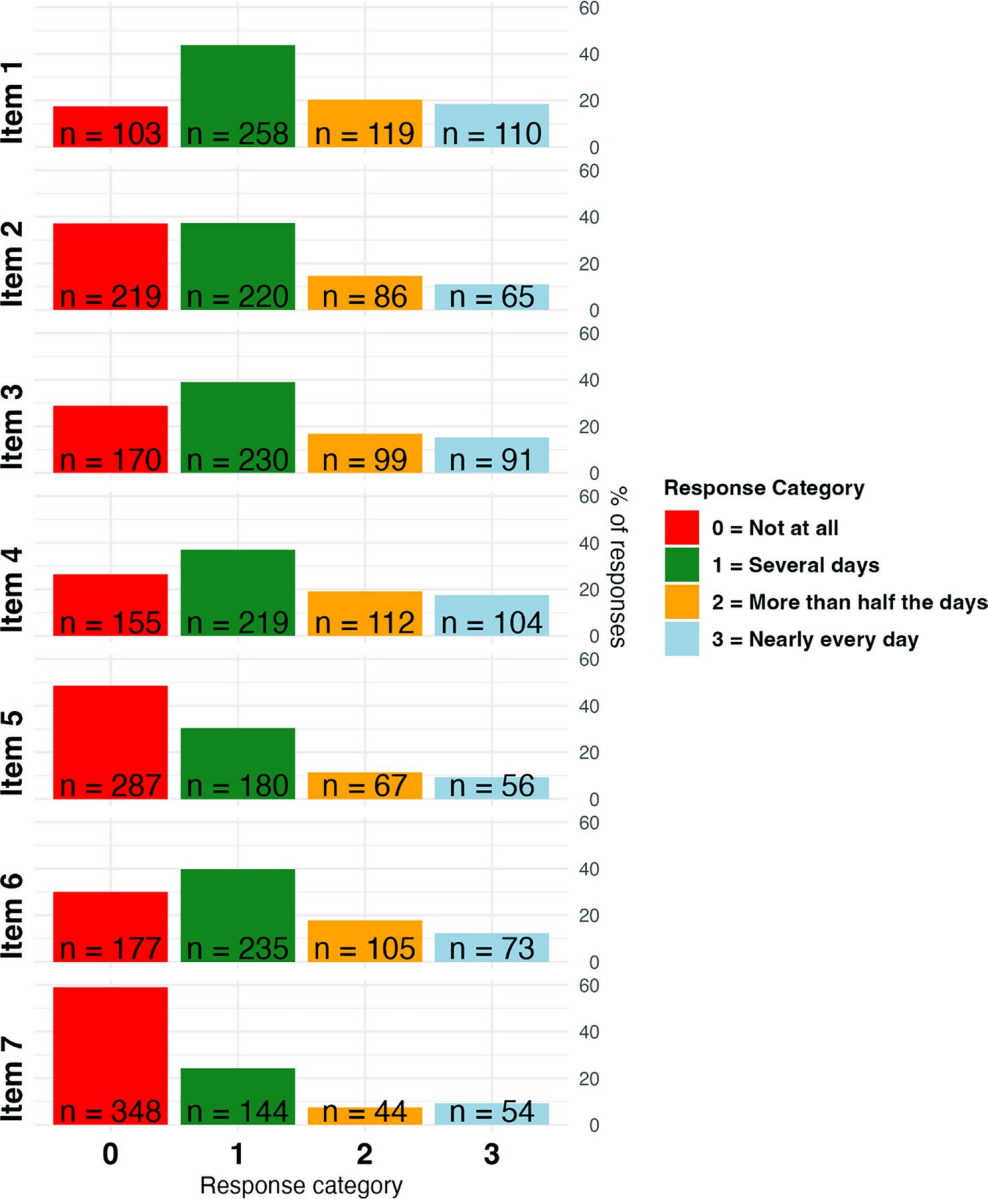
Distribution of overall GAD-7 responses for each response category and each item. GAD-7: The Generalized Anxiety Disorder 7-item scale

Inspection of observed response distributions showed that all response categories were used among the sample, however, indicating a skewed response pattern (Fig. 1).

Results for conditional item fit and item-restscore associations are reported in Table 3, showing that items 1–3 were clearly overfitting and items 5–6 underfitting. The GAD-7 also showed local dependence among items 1–3, with correlations of 0.27 (items 1–2), 0.10 (items 1–3), and 0.26 (items 2–3), all exceeding the relative cutoff of 0.002. The PCAR bootstrap function provided a cutoff for the maximum eigenvalue of 1.45 for the GAD-7, while the observed largest PCAR eigenvalue was 1.92, likewise failing to support unidimensionality. Confirmatory factor analysis of the one-factor structure yielded empirical fit indices of SRMR = 0.043, RMSEA = 0.089, and CFI = 0.992. When compared against the dynamic cutoffs, these indices indicated substantial misfit for the one-factor model as SRMR and RMSEA exceeded, and CFI fell below, the dynamic cutoffs at all levels (0–3). (Table 4).

**Table 3:** Conditional item infit and item-restscore statistics for the GAD-7, GAD 1–3 and GAD 5–7

		Item fit statistics			Item restscore			
	Relative location	Infit MSQ	Infit thresholds	Infit difference	Observed value	Model expected value	Difference	BH adjusted *p*-value
**GAD-7**								
Item 1	0.07	0.721	0.857, 1.159	**-0.162**	0.78	0.65	**0.13**	**0.000**
Item 2	0.93	0.64	0.872. 1.153	**-0.232**	0.79	0.64	**0.15**	**0.000**
Item 3	0.53	0.7	0.850. 1.147	**-0.150**	0.78	0.65	**0.13**	**0.000**
Item 4	0.36	0.86	0.824, 1.132	No misfit	0.72	0.65	**0.07**	**0.004**
Item 5	1.25	1.586	0.866, 1.128	**0.458**	0.42	0.64	**-0.22**	**0.000**
Item 6	0.69	1.365	0.878, 1.152	**0.213**	0.49	0.64	**-0.15**	**0.000**
Item 7	1.47	1.169	0.850, 1.199	No misfit	0.60	0.65	-0.05	0.113
**GAD 1–3**								
Item 1	0.15	1.061	0.860, 1.135	No misfit	0.85	0.86	0.01	0.651
Item 2	1.87	0.845	0.826, 1.112	No misfit	0.90	0.86	0.04	0.066
Item 3	1.02	1.108	0.881, 1.12	No misfit	0.85	0.86	0.01	0.651
**GAD 5–7**								
Item 5	0.55	1.045	0.866, 1.144	No misfit	0.43	0.46	0.03	0.911
Item 6	-0.08	0.953	0.906, 1.107	No misfit	0.48	0.44	0.04	0.911
Item 7	0.88	1.05	0.885, 1.110	No misfit	0.47	0.48	0.01	0.925

MSQ values are based on conditional calculations (*n* = 590 complete cases). Simulation-based thresholds from 300 simulated datasets. Misfitting items are highlighted in Bold. GAD-7: The Generalized Anxiety Disorder 7-item scale. GAD 1–3: Items 1–3 from the original GAD-7. GAD 5–7: Items 5–7 from the original GAD-7 with the highest response categories merged. BH = Benjamini-Hochberg

**Fig. 2:**
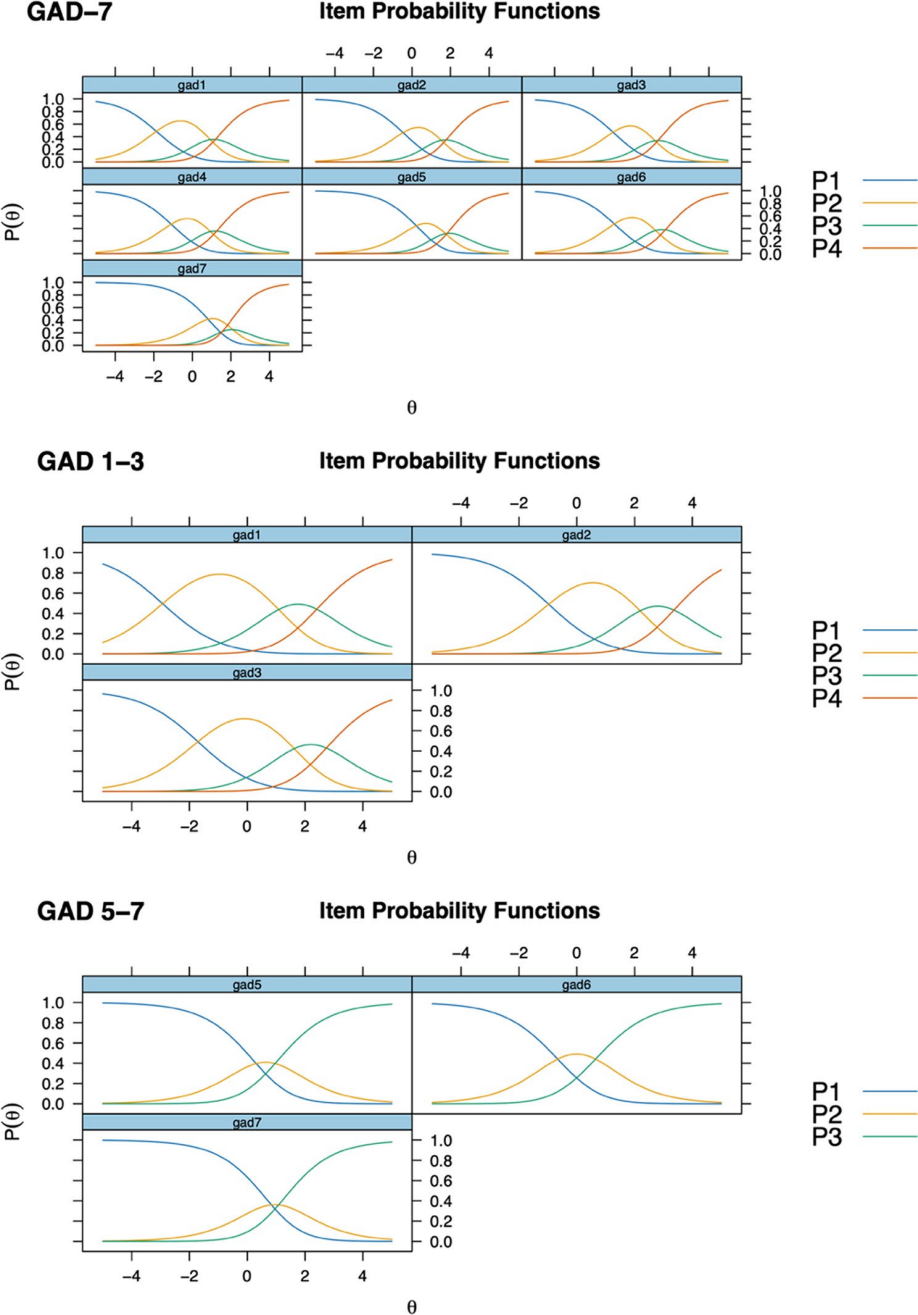
Item probability curves for GAD-7 (upper), GAD items 1–3 (middle), and GAD items 5–7 (lower). Probability of each response category (y-axis) given an individuals’ location on the latent continuum (x-axis). P1 = “Not at all”, P2 = “Several days”, P3 = “More than half the days”, P4 = “Nearly every day”. GAD-7: The Generalized Anxiety Disorder 7-item scale. GAD 1–3: Items 1–3 from the original GAD-7. GAD 5–7: Items 5–7 from the original GAD-7 with the highest response categories merged

The second highest response category (“More than half the days”) of the GAD-7 was never the most likely response at any point on the latent continuum (Fig. 2). Additionally, item 7 had disordered thresholds for thresholds 2 and 3.

For all DIF analyses, response distributions were examined separately for each group and confirmed that there were responses in each category for all items. The GAD-7 showed problematic DIF in terms of average item location for sex at 0.731 logits for item 5, indicating a much higher difficulty for females (0.695, *n* = 411) than males (–0.037, *n* = 179). All other items functioned similarly for both sexes with average item location differences ranging between 0.078 and 0.361. When examining measurement invariance across age, significant DIF was detected at 0.537 logits for item 4 for participants ≤ 18 years (–0.019, *n* = 148) compared to > 18 years (–0.556, *n* = 442). DIF was also detected for item 6 at 0.771 logits for participants ≤ 18 years (–0.628, *n* = 148) compared to > 18 years (0.143, *n* = 442). All other items functioned similarly for different age groups with average location differences ranging from 0.074 to 0.454.

### Iterative process of exploratory scale refinement

Below, findings are reported for the two abbreviated versions (GAD 1–3, GAD 5–7) which emerged from the iterative process of scale refinement prompted by shortcomings revealed in the initial analysis of the GAD-7.

**Fig. 3:**
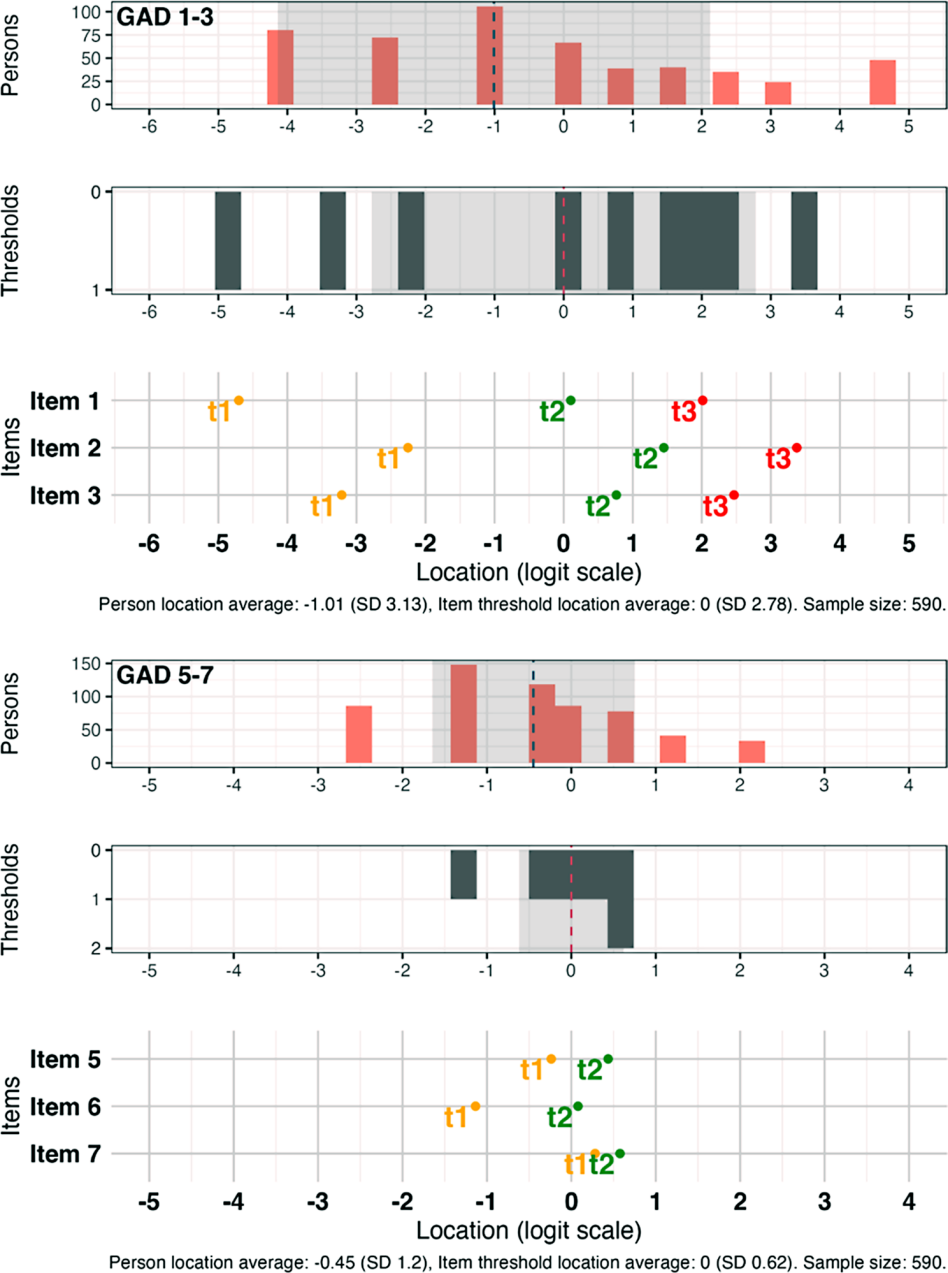
Person-Item Map of GAD 1–3 (upper) and GAD 5–7 (lower). From top to bottom: Person locations histogram, inverted item threshold locations histogram, Individual item threshold locations/Item hierarchy plot. Vertical dotted lines: Mean values. Light grey area: Standard deviation of means for person and item threshold locations, respectively. t1 (yellow): Threshold between category 0 (“Not at all”) and category 1 (“Several days”). t2 (green): Threshold between category 1 (“Several days”) and category 2 (“More than half the days”). t3 (red): Threshold between category 2 (“More than half the days”) and category 3 (“Nearly every day”). GAD 1–3: Items 1–3 from the original Generalized Anxiety Disorder 7-item scale. GAD 5–7: Items 5–7 from the original Generalized Anxiety Disorder 7-item scale with the highest response categories merged

As a first step of exploratory scale refinement, underfitting items were removed one by one from the GAD-7. However, neither removing only item 5, only item 6, nor both of these underfitting items produced results indicative of support for unidimensionality. Analysing item fit for the remaining items 1–4 and 7 resulted in underfit for items 4 and 7. Removing either item 4 or item 7 still resulted in misfit in the item not removed (4 or 7). Only when items 4–7 were all removed, thereby keeping items 1–3 (henceforth called GAD 1–3), did the analyses support unidimensionality based on conditional infit and item-restscore (Table 3). GAD 1–3 showed no local dependence. Also, the simulation-based PCAR cutoff for the maximum eigenvalue was 1.63, and the largest eigenvalue of the GAD 1–3 was 1.61, thus providing support for unidimensionality. GAD 1–3 exhibited adequate response category functioning (Fig. 2); however, a notably large gap between thresholds 1 and 2 was observed for most items, as visualised in the hierarchy plot in Fig. 3. Average item locations indicated no DIF between females and males or across age subgroups.

A second revision of the scale was explored in the opposite direction, removing overfitting items. In this case, only when all items 1–4 were removed, thereby keeping items 5–7, did the results support unidimensionality based on conditional infit and item-restscore (Table 3). Items 5–7 showed no local dependence. The largest eigenvalue of the PCAR was 1.53 and the simulated cutoff was 1.60, also supporting unidimensionality. However, response category functioning analyses indicated problems for items 5 and 7: the second highest response category (“More than half the days”) was never the most likely response at any point on the latent continuum (Fig. 2) and item 7 had disordered thresholds for thresholds 2 and 3. In an effort to achieve satisfactory item probability curves and ordered thresholds, several alternatives of merged response categories were tested. After merging the highest response categories (i.e. 2 and 3) for items 5–7, the revised scale (henceforth called GAD 5–7) demonstrated adequate response category functioning as well as unidimensionality. Average item location indicated no DIF between females and males or across any age subgroups for this revised scale. That said, regarding sex, GAD 5–7 showed item location averages close to the cutoff for item 5 (0.477 logits; females 0.235, males − 0.424) and item 6 (0.463 logits; females − 0.654, males − 0.191). For age, item 7 was near the cutoff for problematic DIF (0.498 logits; ≤20 years 0.742, > 20 years 0.243).

One-factor CFA was not performed for the GAD 1–3 or GAD 5–7 separately as a CFA with only three items would yield perfect but uninformative global fit indices, which do not allow for meaningful evaluation of model fit [54]. However, a 2-factor model CFA was tested combining these abbreviated scales. Only level 0 dynamic cutoffs could be computed with sensitivity > 50% (i.e., the minimum threshold for cutoffs to be considered reliable). Comparing the empirical SRMR (0.031), RMSEA (0.067) and CFI (0.997) to the level 0 dynamic cutoffs did not show support for the two-factor model, although the empirical fit indices showed marginal improvement over those of the one-factor model. Inspection of modification indices for the two-factor model showed no substantial misspecifications, with all values below 10. The largest indices indicated minor residual correlations (e.g., items 2 and 6, and items 5 and 6) and a possible cross-loading of item 7, reflecting slight item overlap. Taken together, the CFA results did not support either a one- or two-factor structure of the GAD-7.

**Table 4:** Confirmatory factor analysis fit indices: empirical values and dynamic cutoffs

	SRMR^c^	RMSEA^d^	CFI^e^
**One-factor model ** **GAD-7** ^**a**^			
Empirical value	0.043	0.089	0.992
Dynamic level^h^ 0 cutoff	0.023^f^	0.032^f^	0.999^f^
Dynamic level^h^ 1 cutoff	0.031^g^	0.054^g^	0.997^g^
Dynamic level^h^ 2 cutoff	0.036^g^	0.074^g^	0.994^g^
Dynamic level^h^ 3 cutoff	0.037^g^	0.081^g^	0.994^g^
**Two-factor model** **GAD 1-3 + GAD 5-7** ^**b**^			
Empirical value	0.031	0.067	0.997
Dynamic level^h^ 0 cutoff	0.023^f^	0.04^f^	0.999^f^

^a^GAD-7: The Generalized Anxiety Disorder 7-item scale

^b^GAD 1-3: Items 1-3 from the original GAD-7. GAD 5-7: Items 5-7 from the original GAD-7 with merged response categories

^c^SRMR = Standardized Root Mean Square Residuals, empirical values should be smaller than cutoffs

^d^RMSEA = Root Mean Square Error of Approximation, empirical values should be smaller than cutoffs

^e^CFI = Comparative Fit Index, empirical values should be larger than cutoffs

^f^95% specificity

^g^95% sensitivity: % of hypothetically misspecified models correctly identified by cutoff in the dynamic fit index simulation

^h^Level-0 corresponds to the anticipated fit index values if the fitted model were the exact underlying population model. The Level-1 row corresponds to the anticipated fit index values if the fitted model omitted 0.30 residual correlations between approximately 1/3 of item pairs. The Level-2 row corresponds to the anticipated fit index values if the fitted model omitted 0.30 residual correlations between approximately 2/3 of item pairs. The Level-3 row corresponds to the anticipated fit index values if the fitted model omitted 0.30 residual correlations between all item pairs. Only level 0 cutoffs are provided for the two-factor model as cutoffs are suppressed when sensitivity is <50%

Figure 3 shows the targeting properties of GAD 1–3 and GAD 5–7, and the improved separation of item thresholds in the revised scales after resolving dimensionality issues and merging response categories. For the two scales, the targeting figure indicates an overall good spread in terms of person locations in the sample. However, item threshold locations do not completely cover the ability of the sample for either scale, indicating that not all participants are targeted by the items. For GAD 1–3, the person location average was − 1.01 (SD 3.13) and the item threshold location average was 0 (SD 2.78). GAD 5–7 had a person location average of -0.45 (SD 1.20) and an item threshold location average of 0 (SD 0.62).

GAD 1–3 demonstrated acceptable to good reliability across indices. The Person Separation Index (PSI = 0.76) indicated acceptable precision in distinguishing between respondents, while both the EAP reliability (0.856) and the RMU estimate (0.849, 95% HDCI = 0.831–0.868) reflected good overall measurement precision and stability of person estimates. With merged response categories, GAD 5–7 demonstrated poor reliability across indices, with a PSI of 0.050, EAP of 0.561 and RMU of 0.550 (95% HDCI = 0.503–0.598).

#### Stability of item fit results

For GAD 1–3, the 10-fold cross-validation revealed that item 2 showed overfit in 90% of folds and item 3 showed underfit in 20% of folds, while item 1 showed stable fit within adaptive cutoffs across all folds. Bootstrapped item-restscore analysis for GAD 1–3 indicated that item 2 showed overfit in 46% of bootstrap iterations. For GAD 5–7, the cross-validation showed fully consistent results with no misfit detected in any fold for any item. Furthermore, bootstrapped item-restscore analysis indicated that no item showed misfit in more than 5% of bootstrap iterations, further supporting the stability of item fit for GAD 5–7. The detailed results are found in Additional File 3.

## Discussion

This psychometric study addressed the current scarcity of studies investigating the psychometric properties of the GAD-7 among adolescents and young adults (AYAs) in a Swedish context, as well as questions regarding the structural validity of the scale. The study comprised two phases: a confirmatory psychometric evaluation of the GAD-7, and an exploratory, iterative refinement process aimed at identifying candidate abbreviated scales, the latter of which should be interpreted with caution until further studied.

Overall, the results indicate that in the current sample, the GAD-7 could not be considered unidimensional and the scale did not fit the Rasch measurement model or the CFA model. While the current findings should be strengthened by evaluations in other samples, they are supported by previous psychometric assessments of the GAD-7 in other European samples of AYAs [10, 11], which have detected problems with the one-factor model fit. The use of the sum score has also been criticized in psychometric research on adult clinical [55] as well as primary care [56] samples. At the same time, these results contradict many prior studies, which have found the GAD-7 to be unidimensional and a valid measure for detecting anxiety disorders [9]. However, previous research has largely relied on rule-of-thumb cutoffs, which have been documented to be inappropriate and pose a risk of misleading results [13, 14].

This Rasch analysis of the GAD-7 detected underfitting items (items 5 and 6), which can be interpreted as the items being poorly formulated or ambiguous [20]. Scrutinizing the misfitting items, they are both double-barrelled, a feature recognised as a potentially serious error in item construction [57]. Revising these items could reduce ambiguity, which might improve item fit. It is also possible that the underfit is due to multidimensionality, indicating that these items may be measuring a separate construct compared to items 1–3. However, the substantive meaning of these item clusters warrants further examination in future research, including criterion validity analyses that were beyond the scope of this study.

The current study undertook an exploratory, iterative process of scale refinement aimed at identifying alternative item combinations that met the validity prerequisites within the current sample. As a result, GAD 1–3 (comprising the first three items of the original scale) was identified as an abbreviated scale that fit the Rasch model. However, the conducted analyses provide little information about the content validity of this candidate scale. The removal of a majority of items from the original GAD-7 raises the question of whether it continues to assess the same underlying construct as intended by the full version. A closer examination of the content in the remaining items also suggests potential limitations in the ability of the GAD 1–3 to distinguish between worry and anxiety disorders. To ensure that GAD 1–3 adequately captures anxiety symptom severity, criterion validity analyses are warranted. Specifically, scores yielded by this scale should be examined in relation to clinical diagnostic interviews - the gold standard for assessing anxiety disorders - as well as other established severity measures.

Although such analyses are recommended for future research, similar investigations have already been conducted for another abbreviated version of the GAD-7 – the GAD-2 [7], which consists of the first two items of the original scale. Like GAD 1–3, it assesses two core characteristics of anxiety disorders, namely anxiousness and uncontrollable worry [58]. Kroenke et al. found the GAD-2 to function similarly to the full GAD-7 in a clinical sample, thereby suggesting that both scales may be equally effective when screening for anxiety disorders. A 2016 systematic review, which included studies validating the GAD-2 against gold standard diagnosis, also concluded that the GAD-2 demonstrates acceptable psychometric properties for identifying GAD, using a cutoff score of three points [59]. Recently, a Swedish study compared the diagnostic accuracy and internal consistency of the GAD-2 and GAD-7 in a clinical sample [60]. The findings of this study likewise supported the GAD-2 as a viable option for assessing anxiety symptom severity, while minimizing respondent burden. However, both studies have pointed out that further validation studies of the GAD-2 are warranted. While GAD 1–3 needs to be studied in further research, it could potentially be a more suitable short-form scale than the GAD-2, particularly in AYAs. Although items 2 and 3 both assess worry, Todorović and colleagues have considered them to evaluate different and clinically important aspects relevant when assessing anxiety [11], which could support the use of GAD 1–3 in favour of the GAD-2.

While GAD 1–3 demonstrated acceptable to good reliability, its brevity inevitably limits its reliability. Although it holds potential as a screening tool at the group level, results of the reliability assessment indicate limited usefulness for evaluating change at the individual level, particularly given that the PSI only reached an acceptable level. This too points to the fact that GAD 1–3 may be a better alternative than the GAD-2, as a measurement tool comprising even fewer items will inevitably be less reliable [61].

Response category functioning analyses in the current study revealed a large gap between thresholds 1 and 2 and scale-to-sample targeting showed that many participants were located within this gap on the logit scale. These findings are similar to previous research conducted on the PHQ-4 [62], which includes the GAD-2 and uses the same response categories. To enhance the reliability of GAD 1–3, future research should consider adding a response category between the existing categories 0 (“Not at all”) and 1 (“Several days”) to improve reliability as well as targeting properties. In a recent evaluation of a Swedish school survey for 15–18 year olds [63, 64], the response category “One or two days” was added for the GAD-2 with psychometric analyses indicating excellent separation of response category thresholds and improved targeting.

The iterative process in this study also identified that items 5–7 from the original scale fit the Rasch model after merging the highest response categories (“More than half the days” and “Nearly every day”). Before merging, the second highest response category was never the most likely response at any point on the latent trait continuum (Fig. 2) and item 7 had disordered thresholds for thresholds 2 and 3. These findings are similar to previous studies [11, 55, 65], one of which also merged the highest response categories, resulting in improved response category functioning [11]. However, while meeting several validity prerequisites, this short version with merged response categories resulted in poor reliability across all indices. The poor reliability of these items is supported by previous research [55], which also found items 5–7 to differ from the other items. As a result, the practical use of this abbreviated scale is considered limited, given its insufficient precision and stability of the person estimates.

This study should be read with some limitations in mind. First, there is the risk of self-selection bias, as it is likely that participation has been more common among individuals with experiences of anxiety, or mental ill-health in general [66]. However, results showed a skewed distribution toward lower levels of anxiety. The proportion of participants born outside Sweden was low compared to the general population in this age group [67], which limits the representativeness of the sample and the ability to generalize findings to the broader Swedish AYA population. Furthermore, the findings are specific to this community-based sample of AYAs and may not generalize to clinical populations or other contexts in which the GAD-7 is commonly used.

Another limitation is that this study could not investigate DIF for different subgroups of symptom severity. While the aim was to do so by splitting participants into two groups using their median sum scores, inspection of overall responses showed that there were too few responses for each response category in each subgroup to assess DIF in a meaningful way. Future research should therefore employ larger sample sizes to ensure that the abbreviated candidate scales are invariant across subgroups defined by symptom severity.

Finally, the exploratory, iterative refinement process was conducted within the same dataset used for the initial evaluation of the GAD-7, which carries a risk of overfitting. The abbreviated candidate scales may therefore fit this specific sample better than they would in an independent sample, further underscoring the need for external validation before any conclusions can be drawn about their broader utility. However, cross-validation and bootstrapped item-restscore analyses did provide some reassurance regarding the stability of item fit findings for GAD 5–7, for which no misfit was detected in any fold and no item showed misfit in more than 5% of bootstrap iterations. For GAD 1–3, item 1 showed stable fit across all folds, while item 2 showed overfit in 90% of cross-validation folds and 46% of bootstrap iterations, and item 3 showed underfit in 20% of cross-validation folds. These findings warrant attention in future validation studies.

A strength of this study is that it applied modern psychometric methods, many of which have been proposed as superior to traditional techniques [13, 15, 18]. However, it is important to acknowledge that some methods, such as the RMU reliability estimate and the simulation-based approach for determining PCAR cutoffs, have not yet been extensively tested under a wide range of conditions [41]. Further research is therefore needed to establish the robustness of these approaches. To mitigate this limitation, multiple complementary analyses were conducted to avoid reliance on any single method.

## Conclusions

Neither Rasch nor confirmatory factor analysis confirmed the unidimensionality of the GAD-7 among AYAs in this Swedish sample, which calls into question the use of the GAD-7 sum score in this context. Exploratory scale refinement analyses found the first three items of the scale, GAD 1–3, to fit the Rasch model in the current sample. Nonetheless, the relatively low reliability of the abbreviated scale must be acknowledged, which limits its use for individual assessment. Future research should explore the potential benefits of adding a response category between the first and second options to enhance both reliability and targeting. Moreover, the abbreviated scale warrants further evaluation, including criterion validity assessment through comparison with clinical diagnostic interviews and established severity measures. The current reports of declining mental health among AYAs along with the lack of comprehensive assessments of anxiety in this group highlight the need for further research to identify a scale capable of validly and reliably assess anxiety symptom severity within this population.

## Supplementary Information

Below is the link to the electronic supplementary material.

Supplementary Material 1

Supplementary Material 2

Supplementary Material 3

## Data Availability

The datasets generated and analysed during the current study are not publicly available due to ethical restrictions according to Swedish law agreed upon by researchers and participants. Sharing of pseudonymized data would be contingent upon obtaining additional ethics approval for data sharing.

## Abbreviations

GAD-7: The Generalized Anxiety Disorder 7-item scale
DSM: The American Psychiatric Association’s Diagnostic and Statistical Manual of Mental Disorders
AYAs: Adolescents and Young Adults
GAD: Generalized Anxiety Disorder
RMT: Rasch Measurement Theory
CTT: Classical Test Theory
UPIC: Young people’s mental health in focus
PCM: Partial Credit Model
MSQ: Weighted Mean Squares
PCAR: Principal Components Analysis of Rasch model residuals
CFA: Confirmatory Factor Analysis
WLSMV: Weighted Least Squares Mean and Variance Adjusted
CFI: Comparative Fit Index
RMSEA: Root Mean Square Error of Approximation
SRMR: Standardized Root Mean Square Residual
DIF: Differential Item Functioning
PSI: Person Separation Index
EAP: Expected A Posteriori
RMU: Relative Measurement Uncertainty
HDCI: Highest Density Continuous Interval
SD: Standard Deviation
