# Supplementary material for: Psychometric assessment and exploratory scale refinement of the Generalized Anxiety Disorder 7-item scale among adolescents and young adults in a Swedish context
Source: BMC Psychiatry. 2026 Jul 23;26:564. doi: 10.1186/s12888-026-08423-0 (PMC13397768; doi:10.1186/s12888-026-08423-0)
Supplement: Supplementary file 1 — Supplementary Material 1 [file 12888_2026_8423_MOESM1_ESM.docx]

Additional file 1: Person fit statistics and ordinal sum score responses

### **Person fit**

Of all participants, 10.5% (n = 62) were flagged by the *U*^3^ metric as misfitting the Rasch model when assessing the GAD-7. For GAD 1-3, 9.8% (n = 58) participants were misfitting, and for GAD 5-7 10.5% (*n* = 62).

### **Ordinal sum score responses**

The figure below displays the distribution of GAD-7, GAD 1-3 and GAD 5-7 ordinal sum scores. The participants had a GAD-7 sum score range of 0-21 points with a median of 7 points (Q1-Q3:7-11). Of the sample, 4.92 % had 0 points, and 1.19 % got the maximum of 21 points. According to suggested cutoffs for the GAD-7, 35.3 % (n = 208) of the sample had sum scores corresponding to mild anxiety, 19.7% (n = 116) to moderate anxiety and 11.0% (n = 65) to severe anxiety. Keeping only items 1-3 resulted in a GAD 1-3 sum score range of 0-9 points with a median of 3 points (Q1-Q3: 1-5). Keeping only items 5-7 and merging the highest response categories resulted in a GAD 5-7 raw sum score range of 0-6 points with a median of 2 points (Q1-Q3: 1-4).


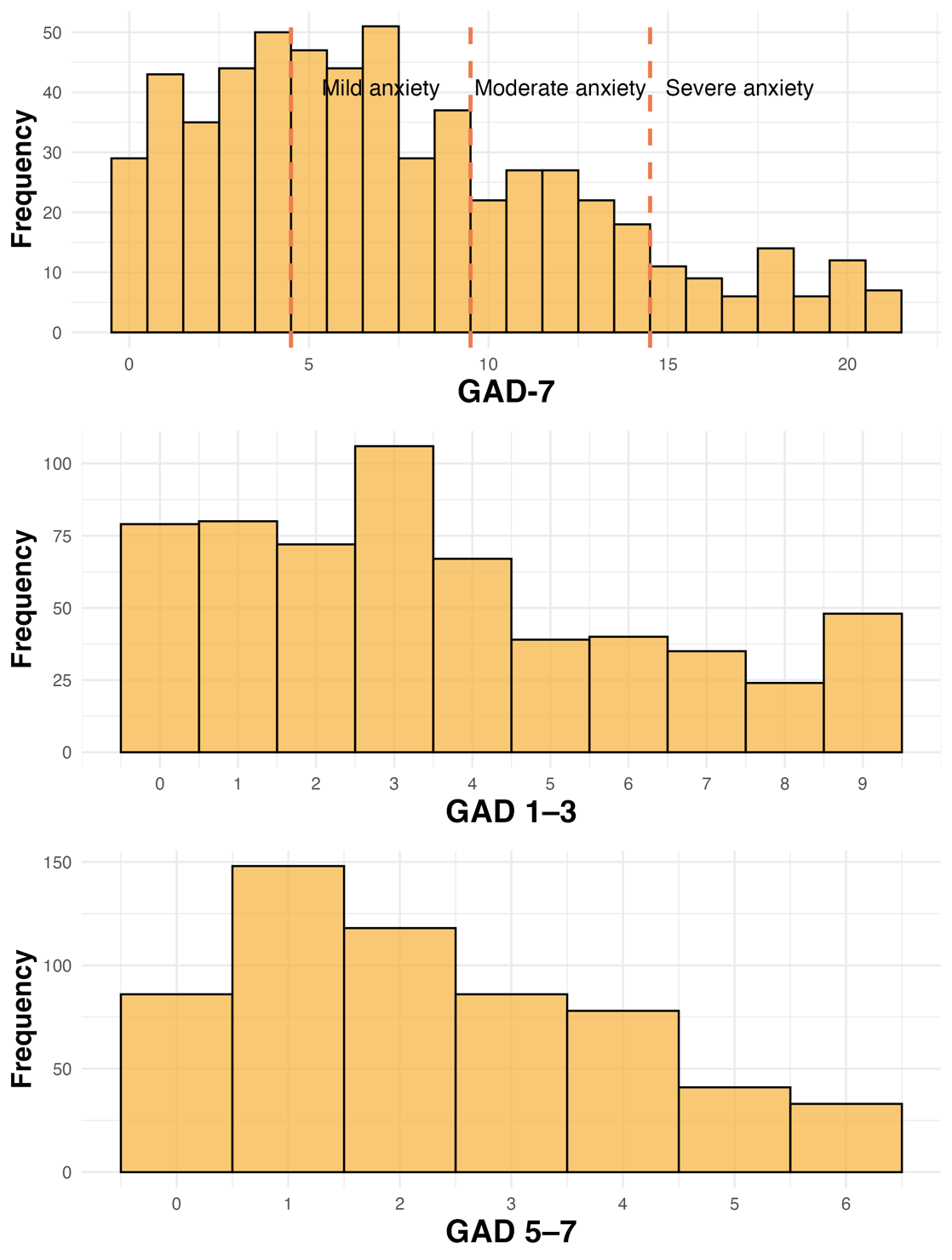


Distribution of ordinal sum scores for the GAD-7, GAD 1-3 and GAD 5-7. From top to bottom: GAD-7: The Generalized Anxiety Disorder 7-item scale, possible sum score range 0-21, presented with suggested interpretational cutoffs (5-9 points = mild anxiety, 10-14 points = moderate anxiety, 15-21 points = severe anxiety). Mild anxiety = 5-9 points**;** Moderate anxiety = 10-14 points; Severe anxiety = 15-21 points. GAD 1-3: Items 1-3 from the original GAD-7, possible sum score range 0-9. GAD 5-7: Items 5-7 from the original GAD-7 with the highest response categories merged, possible sum score range 0-6.
