# Supplementary material for: Psychometric assessment and exploratory scale refinement of the Generalized Anxiety Disorder 7-item scale among adolescents and young adults in a Swedish context
Source: BMC Psychiatry. 2026 Jul 23;26:564. doi: 10.1186/s12888-026-08423-0 (PMC13397768; doi:10.1186/s12888-026-08423-0)
Supplement: Supplementary file 2 — Supplementary Material 2 [file 12888_2026_8423_MOESM2_ESM.docx]

## Additional file 2: Item parameters and ordinal sum score to interval table

| Item parameters | | | | |
| --- | --- | --- | --- | --- |
|  | **Threshold 1** | **Threshold 2** | **Threshold 3** | **Item location** |
| **GAD-7** |  |  |  |  |
| **Item 1** | -2.67 | 0.20 | 0.41 | -0.69 |
| **Item 2** | -1.21 | 0.75 | 0.98 | 0.17 |
| **Item 3** | -1.72 | 0.47 | 0.57 | -0.23 |
| **Item 4** | -1.86 | 0.21 | 0.47 | -0.4 |
| **Item 5** | 0.52 | 0.97 | 1.03 | 0.49 |
| **Item 6** | -1.66 | 0.50 | 0.95 | -0.07 |
| **Item 7** | 0.07 | 1.30 | 0.76 | 0.71 |
|  |  |  |  |  |
| **GAD 1-3** |  |  |  |  |
| **Item 1** | -4.70 | 0.10 | 2.01 | -0.86 |
| **Item 2** | -2.26 | 1.45 | 3.38 | 0.86 |
| **Item 3** | -3.21 | 0.76 | 2.47 | 0 |
|  |  |  |  |  |
| **GAD 5-7** |  |  |  |  |
| **Item 5** | -0.24 | 0.44 |  | 0.1 |
| **Item 6** | -1.14 | 0.08 |  | -0.53 |
| **Item 7** | 0.28 | 0.58 |  | 0.43 |
| Item location is the average of thresholds for each item. GAD-7: The Generalized Anxiety Disorder 7-item scale. GAD 1-3: Items 1-3 from the original GAD-7. GAD 5-7: Items 5-7 from the original GAD-7 with the highest response categories merged. | | | | |

| Corresponding logit score with standard error to every ordinal sum score for GAD 1-3 and GAD 5-7. | | | |
| --- | --- | --- | --- |
|  | **Ordinal sum score** | **Logit score** | **Logit standard error** |
| **GAD 1-3** |  |  |  |
|  | 0 | -5.882 | 0.909 |
|  | 1 | -4.068 | 1.206 |
|  | 2 | -2.674 | 1.226 |
|  | 3 | -1.113 | 1.130 |
|  | 4 | 0.160 | 1.018 |
|  | 5 | 0.984 | 0.928 |
|  | 6 | 1.692 | 0.891 |
|  | 7 | 2.409 | 0.919 |
|  | 8 | 3.266 | 0.956 |
|  | 9 | 4.692 | 0.761 |
|  |  |  |  |
| **GAD 5-7** |  |  |  |
|  | 0 | -2.399 | 0.684 |
|  | 1 | -1.160 | 0.842 |
|  | 2 | -0.486 | 0.816 |
|  | 3 | 0.046 | 0.790 |
|  | 4 | 0.555 | 0.793 |
|  | 5 | 1.154 | 0.784 |
|  | 6 | 2.232 | 0.624 |
| GAD 1-3: Items 1-3 from the original GAD-7. GAD 5-7: Items 5-7 from the original Generalized Anxiety Disorder 7-item scale with the highest response categories merged. | | | |
