# Supplementary material for: Psychometric assessment and exploratory scale refinement of the Generalized Anxiety Disorder 7-item scale among adolescents and young adults in a Swedish context
Source: BMC Psychiatry. 2026 Jul 23;26:564. doi: 10.1186/s12888-026-08423-0 (PMC13397768; doi:10.1186/s12888-026-08423-0)
Supplement: Supplementary file 3 — Supplementary Material 3 [file 12888_2026_8423_MOESM3_ESM.docx]

Additional file 3: Stability of item fit results

| Cross-validation of conditional item infit for abbreviated scales | | | | |
| --- | --- | --- | --- | --- |
|  | **Lower cutoff** | **Lowest infit MSQ** | **Highest infit MSQ** | **Upper cutoff** |
| **GAD 1-3** | | | | |
| Item 1 | 0.875 | 1.024 | 1.082 | 1.125 |
| Item 2 | 0.871 | 0.818 | 0.856 | 1.112 |
| Item 3 | 0.867 | 1.067 | 1.169 | 1.126 |
| **GAD 5-7** | | | | |
| Item 5 | 0.879 | 1.024 | 1.069 | 1.119 |
| Item 6 | 0.904 | 0.945 | 0.958 | 1.117 |
| Item 7 | 0.851 | 1.005 | 1.084 | 1.129 |
| *Note. Infit MSQ values based on conditional estimation using n = 531 cases (10 folds of data from a dataset of 590). Cutoff values based on 300 simulations from each fold of data.* | | | | |


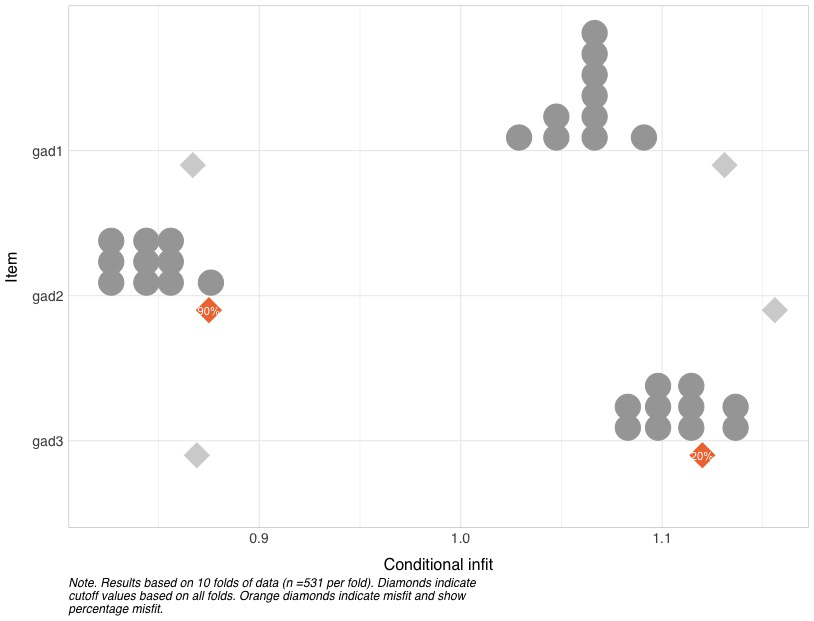


Figure 1. GAD 1-3


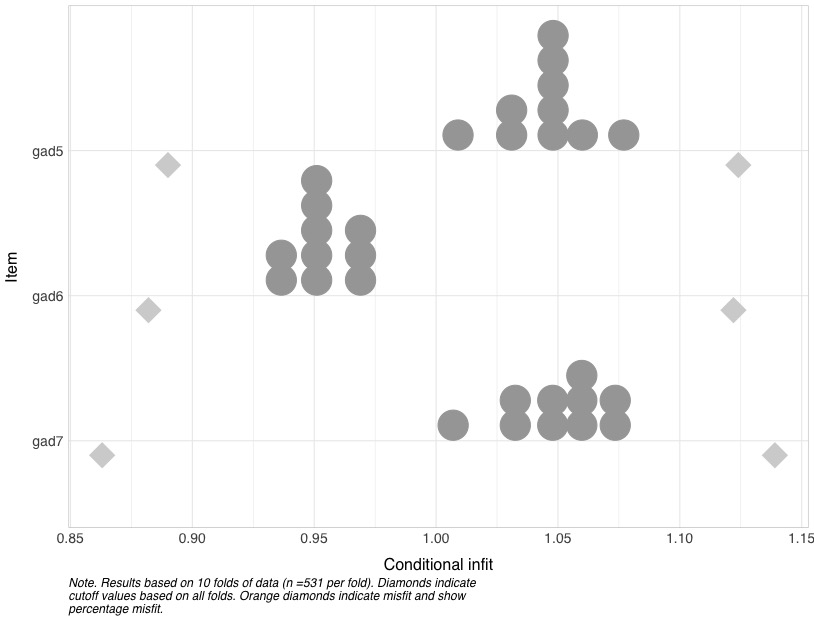


Figure 2. GAD 5-7
